# Mapping Drought Vulnerability in the Chi River Basin, Thailand: A Machine Learning Framework Using H3 Hexagonal Grids and Topographic Variables

**DOI:** 10.3390/s26154760

**Published:** 2026-07-27

**Authors:** Narueset Prasertsri, Patiwat Littidej, Benjamabhorn Pumhirunroj, Donald Slack

**Affiliations:** 1 Department of Geoinformatics, Research Unit of Geoinformatics for Spatial Management, Faculty of Informatics, Mahasarakham University, Maha Sarakham 44150, Thailand; narueset.p@msu.ac.th (N.P.); patiwat.l@msu.ac.th (P.L.); 2 Program in Animal Science, Faculty of Agricultural Technology, Sakon Nakhon Rajabhat University, Sakon Nakhon 47000, Thailand; 3 Department of Civil & Architectural Engineering & Mechanics, University of Arizona, 1209 E. Second St., Tucson, AZ 85721, USA; slackd@arizona.edu

**Keywords:** drought risk mapping, vulnerability assessment, AI and GIS integration, Chi River Basin, Maha Sarakham, H3 hexagonal grid, machine learning, LightGBM, SHAP analysis, topographic controls

## Abstract

**Highlights:**

**What are the main findings?**

- LightGBM achieved AUC = 0.783 (temporal test-set validation) and AUC = 0.714 (spatial holdout) for binary drought detection, with static spatial variables (slope, DEM, TWI, prox2river) dominating SHAP importance (76.1%) over remote sensing indices (23.9%).
- Five-class severity accuracy on the 2024 test set ranged from 71.4% (Very Low) to 25.0% (Severe), limited by small sample sizes for rare classes; the framework is best characterized as a drought risk mapping tool rather than an operational early warning system.

**What are the implications of the main findings?**

- The framework provides a replicable methodology for identifying spatially recurrent drought-prone areas at village-relevant scales (H7 grids ≈ 5.96 km^2^) using publicly available Sentinel-2 data and open-source machine learning tools.
- The methodology is transferable to similar floodplain environments in Southeast Asia, though local re-estimation and independent validation are required.

**Abstract:**

Drought is a recurrent hazard in the Chi River Basin, northeastern Thailand, causing agricultural losses despite its low-lying floodplain setting. This study developed a machine learning framework integrating Sentinel-2 indices (SMI, NDVI, MNDWI, VCI, NDMI) and static spatial variables (DEM, TWI, prox2river, slope) aggregated to H3 hexagonal grids (≈5.96 km^2^) for village-relevant analysis. Drought reports from 1482 observations (2019–2024) were aggregated to 203 grid cells. Three models—Random Forest, XGBoost, and LightGBM—were evaluated using temporal (training: 2019–2023; test: 2024) and spatial holdout validation. LightGBM achieved the best performance with AUC = 0.783 (temporal) and 0.714 (spatial), accuracy = 78.3%, and balanced accuracy = 76.4%. Five-class severity classification showed declining accuracy from 71.4% (Very Low) to 25.0% (Severe), limited by rare event sample sizes. SHAP analysis revealed static topographic variables dominated importance (76.1%) over remote sensing indices (23.9%), with weak individual correlations (|r| < 0.10). The framework is best characterized as a drought risk mapping tool for identifying persistently vulnerable areas rather than an operational early warning system. The methodology is transferable to similar floodplain environments with local re-estimation and validation.

## 1. Introduction

Drought is a complex natural hazard that causes significant agricultural losses, water scarcity, and socioeconomic impacts worldwide [1,2]. Unlike rapid-onset disasters such as floods or storms, drought develops gradually over extended periods, making early detection and prediction particularly challenging [3]. Climate change projections indicate that drought frequency and severity will increase in many regions, particularly in tropical and subtropical areas of Southeast Asia [4,5]. In Thailand, drought events have become increasingly frequent and severe over the past two decades, threatening food security, rural livelihoods, and water resource sustainability [6,7].

The Chi River Basin in northeastern Thailand (Isan region) is particularly vulnerable to drought despite being characterized as a low-lying floodplain area [8,9]. Maha Sarakham Province, located in the heart of the Chi River Basin, experiences recurrent drought events that affect approximately 60–70% of agricultural land during severe years [10,11]. This apparent paradox—frequent drought occurring in a floodplain environment—is attributed to several factors: (1) sandy and sandy loam soils with low water-holding capacity; (2) shallow groundwater tables that are quickly depleted; (3) seasonal rainfall variability with prolonged dry spells; and (4) limited irrigation infrastructure despite proximity to the Chi River [12,13,14]. Previous studies have documented that Maha Sarakham experiences drought conditions in approximately 8 out of 15 years, with estimated agricultural losses exceeding 500 million baht annually [10].

Remote sensing technology has emerged as a powerful tool for drought monitoring, providing synoptic coverage, repeated observations, and consistent measurements of land surface conditions across large areas [15,16]. Various remote sensing indices have been developed to capture different biophysical aspects of drought, including vegetation response (NDVI, VCI), surface water availability (MNDWI), vegetation moisture content (NDMI), and soil moisture conditions (SMI) [17,18,19]. Multi-temporal remote sensing indices have demonstrated utility for drought assessment in the Chi River Basin, with combined indices providing better drought detection accuracy than any single indicator [20]. This finding highlights the importance of integrating multiple satellite-derived parameters to capture the complex, multi-faceted nature of drought conditions in the Chi River floodplain, where meteorological drought rapidly translates into agricultural drought due to poor soil water retention [21,22].

However, most previous studies have focused on pixel-based analyses or aggregated data to administrative boundaries that may not align with the actual spatial units where drought impacts are experienced and response decisions are made [23,24]. In Maha Sarakham Province, village-level drought impacts vary considerably even within the same sub-district due to differences in soil type, elevation, proximity to the Chi River, and access to irrigation [25,26]. This mismatch limits the practical utility of drought assessment models for village-level resource allocation and targeted intervention [27,28].

The hexagonal grid system, particularly Uber’s H3 hierarchical spatial index, offers several advantages over traditional square grids for spatial analysis [29,30]. Hexagonal grids provide uniform adjacency relationships, reduce sampling bias, and more accurately represent circular spatial processes such as drought propagation [31,32]. When aggregated to appropriate resolution levels, hexagonal grids can provide spatial units that correspond approximately to village administrative areas, enabling analysis at the scale where drought response actions are implemented [33,34]. For the Chi River Basin, this study utilizes H3 hexagon level 7 (H7) grid cells (approximately 5.96 km^2^ per cell), which correspond approximately to the average area of village administrative units in Maha Sarakham Province. It should be noted that this correspondence is based on approximate area matching rather than exact boundary alignment; operational use would require translation from H7 cells to village boundaries.

Machine learning methods have shown considerable promise for drought assessment by capturing complex, non-linear relationships between remote sensing indices and drought conditions [35,36]. Ensemble methods such as Random Forest (RF), XGBoost, and LightGBM have demonstrated superior performance compared to traditional statistical approaches, particularly when handling multi-source data with varying scales and distributions [37,38,39]. SHAP (SHapley Additive exPlanations) provides a unified framework for interpreting machine learning predictions by quantifying the contribution of each feature to individual predictions, enabling identification of the most influential predictors and understanding of model decision-making processes [40,41].

This study addresses several critical research gaps: (1) the apparent paradox of frequent drought occurrence in the Chi River floodplain of Maha Sarakham Province remains underexplored using integrated remote sensing and machine learning approaches; (2) the potential of H3 hexagonal grid systems at level 7 (H7) for village-relevant drought assessment has not been systematically evaluated; (3) the relative contribution of remote sensing indices versus static spatial variables to drought risk assessment has not been quantified; and (4) comparative evaluations of different machine learning algorithms for drought assessment using consistent hexagonal grid units are lacking for the Chi River Basin context.

The specific objectives of this study are: (1) to aggregate drought report data and remote sensing indices to H3 hexagon level 7 (H7) grids (approximately 5.96 km^2^ per cell) for village-relevant analysis in Maha Sarakham Province; (2) to evaluate and compare the performance of Random Forest, XGBoost, and LightGBM models for drought risk assessment using both temporal and spatial validation designs; (3) to identify the most important predictors of drought risk using SHAP analysis; (4) to assess the contribution of remote sensing indices versus static spatial variables to predictive accuracy; and (5) to develop a practical framework for village-level drought risk assessment applicable to floodplain environments with similar characteristics.

This research aligns with the emerging field of smart geospatial analytics, which integrates artificial intelligence with geographic information systems for evidence-based environmental management [42]. By combining LightGBM machine learning with H3 hexagonal grid analysis, this study provides a replicable framework for drought risk assessment that addresses the “last mile” challenge of translating regional assessments to actionable village-level information.

The remainder of this paper is organized as follows: Section 2 describes the study area, data sources, and methodology. Section 3 presents the experimental results, including model performance comparisons, SHAP feature importance analysis, and spatial patterns of drought risk. Section 4 discusses the findings in the context of previous research and the Chi River Basin’s unique characteristics. Section 5 concludes the study with recommendations for operational drought management and future research directions.

## 2. Materials and Methods

### 2.1. Study Area: Chi River Basin, Maha Sarakham Province

**Figure 1:**
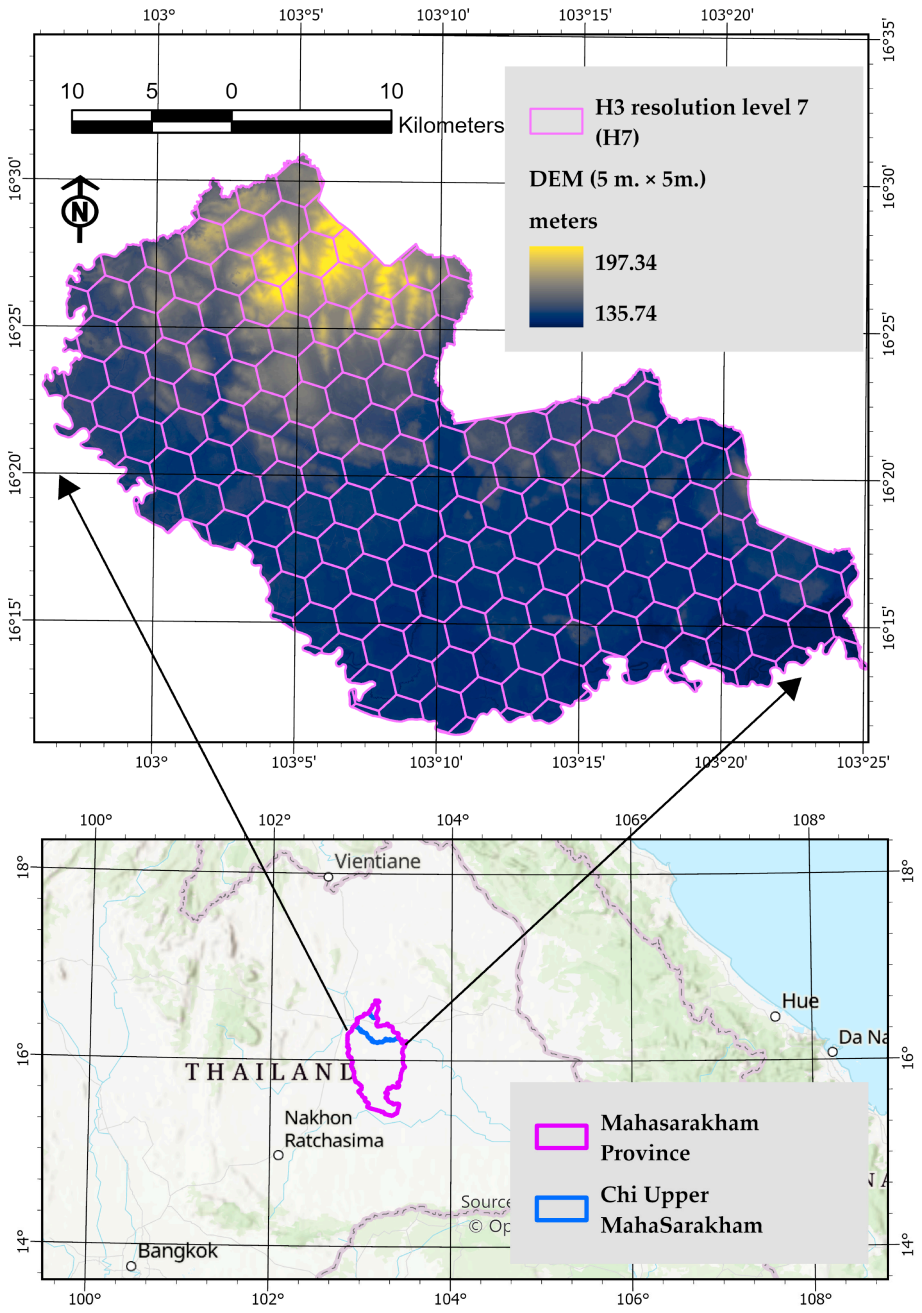
Location map of the study area showing: geographic location of Maha Sarakham Province within northeastern Thailand; digital elevation model (DEM) with 5 m resolution, showing elevations ranging from 135.74 to 197.34 m above sea level; major river networks (Chi River and tributaries shown in blue); and the 203 H3 hexagon level 7 (H7) grid cells (approximately 5.96 km^2^ per cell) used as spatial analysis units.

The study area comprises 203 hexagonal grid cells at H3 resolution level 7 (H7), covering approximately 5.96 km^2^ per cell, totaling approximately 1210 km^2^ of Maha Sarakham Province and surrounding areas in the Chi River Basin, northeastern Thailand. Figure 1 presents the location map of the study area, showing the geographic location of Maha Sarakham Province within northeastern Thailand, a 5 m resolution digital elevation model (DEM) with elevations ranging from 135.74 to 197.34 m above sea level, major river networks (Chi River and its tributaries), and the distribution of the 203 H3 hexagon level 7 (H7) grid cells that serve as the spatial analysis units throughout this study.

Maha Sarakham Province (16°30′ N, 103°15′ E) is characterized by low-lying topography with elevations ranging from 135.74 to 197.34 m above sea level (based on 5 m resolution DEM data), predominantly flat terrain with slopes generally less than 2°, and a dense network of the Chi River and its tributaries [1,2]. Despite this floodplain setting, the province experiences recurrent and severe drought due to three primary factors. First, the region is underlain by the Khorat Plateau geologic formation, characterized by sandy and sandy loam soils (Yasothon soil series, Nam Phong soil series) with very low water-holding capacity (available soil water typically 50–80 mm/m) and high infiltration rates [3,4]. Second, the Chi River is a seasonal river with highly variable flow regimes. During the dry season (November–April), many river sections shrink to less than 20% of wet season width, and some tributaries completely dry up [5,6]. Third, the region experiences pronounced seasonal rainfall variability with a distinct dry season (November–April) and wet season (May–October), and prolonged dry spells are common even during the wet season [7,8].

The H3 hexagonal grid system at resolution level 7 (H7) was selected because each cell of approximately 5.96 km^2^ corresponds approximately to the average area of village administrative units in Maha Sarakham Province [9,10]. This approximate correspondence enables translation of model predictions to village-level information, though exact alignment requires qualification (see Section 4.5). The hexagonal cells provide uniform adjacency (six equidistant neighbors) and reduce spatial bias inherent in square grids, which is particularly important for analyzing drought propagation along the Chi River network [11,12]. The 5 m resolution DEM provides high-precision elevation data, allowing accurate extraction of topographic attributes including slope and hydrological flow patterns at the village scale [13,14].

### 2.2. Drought Report Data and Spatial Aggregation to Hexagonal Grid

Drought report data were collected annually from field surveys conducted by the Maha Sarakham Provincial Agricultural Office and village administrative records for the years 2019, 2020, 2023, and 2024 [13]. Each report was geolocated to the centroid of a 1 km × 1 km administrative reporting unit and recorded with a drought severity score ranging from 0 to 18 points per reporting unit. The scoring system is based on: (1) percentage of agricultural area affected (0–6 points), (2) duration of water shortage in weeks (0–6 points), and (3) number of households reporting water scarcity (0–6 points) [14]. A total of 1482 individual drought reports were compiled across the four-year study period.

To enable spatial analysis at the village level and facilitate integration with remote sensing data, the point-based drought reports were aggregated to the same H3 hexagon level 7 (H7) grid cells using zonal statistics [15]. For each H7 cell and year, the sum of drought severity scores (sum_drought) was calculated, ranging from 0 to 18 points per cell-year. The classification scheme divides the drought counts into five classes based on left-closed, right-open intervals:

Very Low: 0 ≤ sum_drought < 2;

Low: 2 ≤ sum_drought < 4;

Moderate: 4 ≤ sum_drought < 7;

High: 7 ≤ sum_drought < 11;

Severe: 11 ≤ sum_drought ≤ 18.

**Figure 2:**
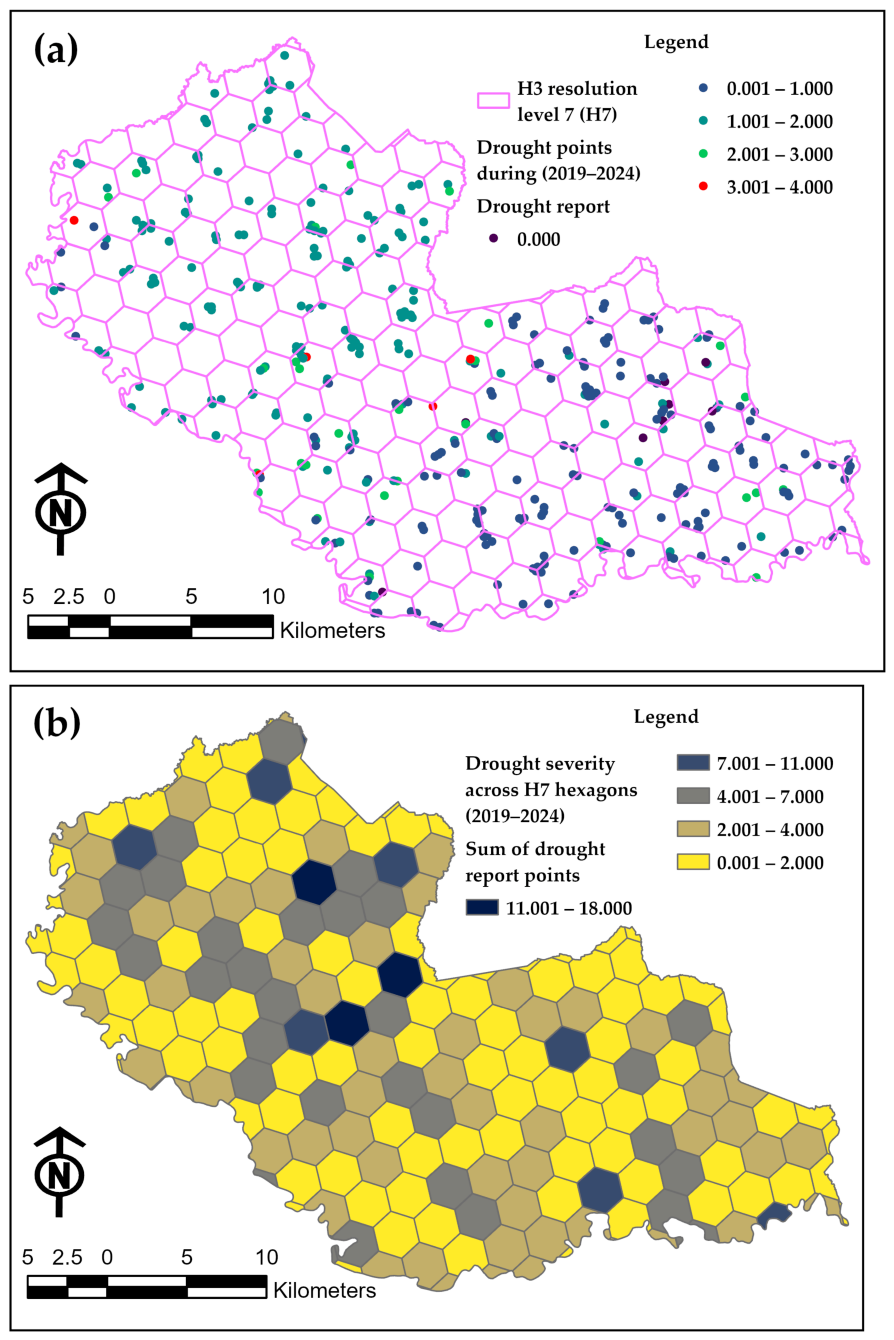
(**a**) Cumulative drought report points (2019, 2020, 2023, 2024) overlaid on H3 hexagon level 7 (H7) grid cells in Maha Sarakham Province. (**b**) Drought count aggregated to H3 hexagon level 7 (H7) grid cells (approximately 5.96 km^2^ per cell), classified into five classes based on sum_drought values.

Cells with sum_drought = 0 are included in the Very Low class. For binary classification, drought conditions are defined as sum_drought > 1 (i.e., any reported drought impact above minimal levels). This threshold was chosen based on operational practice in the study area, where any reported impact above baseline triggers initial response considerations. Figure 2 illustrates the spatial distribution of cumulative drought report points and the aggregated drought count across the H3 hexagon level 7 (H7) grid cells.

### 2.3. Remote Sensing Indices

Five remote sensing indices were calculated from Sentinel-2 imagery [43] to characterize drought conditions from multiple biophysical perspectives [23,24]. Table 1 summarizes the indices, their formulas, theoretical ranges, and drought interpretation. It should be noted that SMI and NDMI are conceptually distinct but operationally similar when calculated from the same bands; SMI typically incorporates additional atmospheric correction, while NDMI is a simpler normalized difference. In this implementation, both were retained to evaluate whether the additional processing in SMI provided unique information.

**Figure 3:**
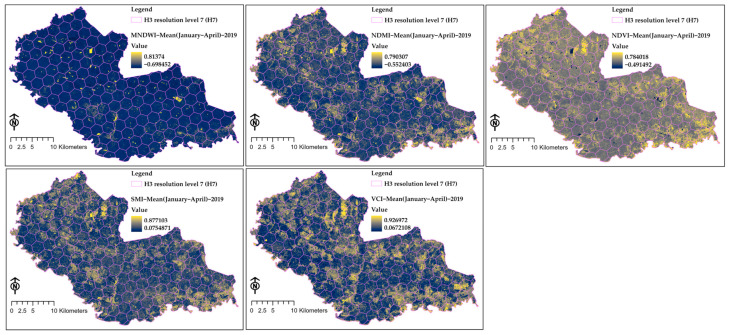
Spatial distribution of remote sensing indices (MNDWI, NDMI, NDVI, SMI, VCI) aggregated to H3 hexagon level 7 (H7) grid cells for the 2019 dry season (January–April).

**Figure 4:**
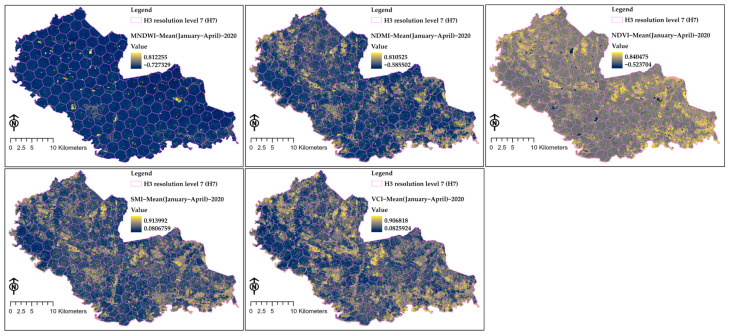
Spatial distribution of remote sensing indices for the 2020 dry season (La Niña year).

**Figure 5:**
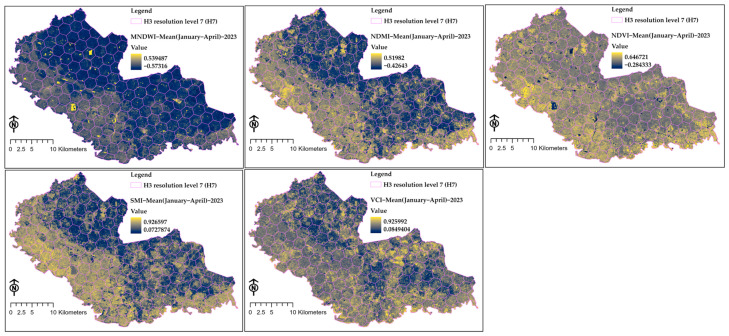
Spatial distribution of remote sensing indices for the 2023 dry season (El Niño year).

**Figure 6:**
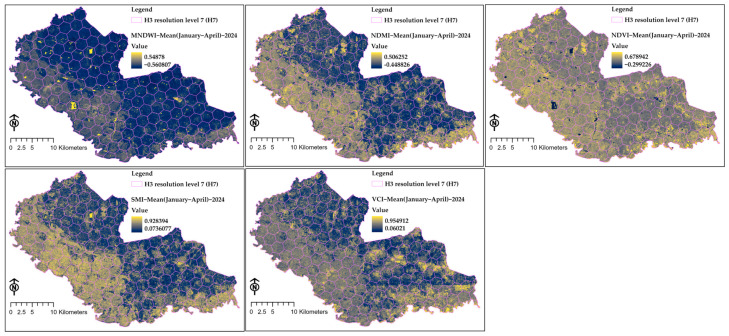
Spatial distribution of remote sensing indices for the 2024 dry season (transition year).

Figure 3, Figure 4, Figure 5 and Figure 6 present the spatial distribution of remote sensing indices for the 2019, 2020, 2023, and 2024 dry seasons, respectively.

### 2.4. Static Spatial Variables

**Figure 7:**
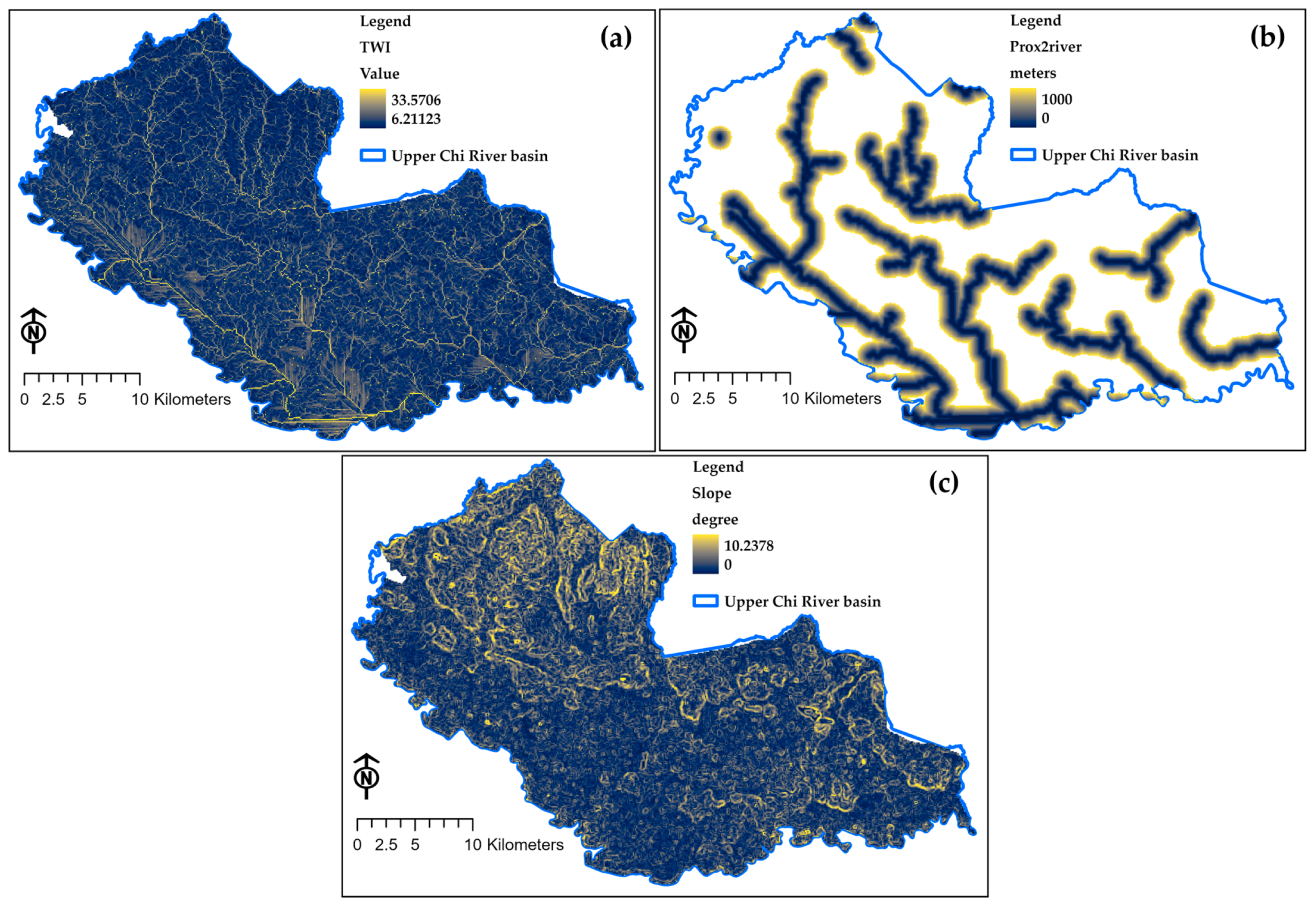
Spatial distribution of static spatial variables aggregated to H3 hexagon level 7 (H7) grid cells: (**a**) topographic Wetness Index (TWI); (**b**) proximity to streams (prox2river); and (**c**) slope.

Four static spatial variables were derived from the 5 m resolution digital elevation model (DEM): slope, DEM, Topographic Wetness Index (TWI), and proximity to streams (prox2river) (see Figure 7). These variables capture topographic and hydrological controls on drought susceptibility in the Chi River floodplain [25,26].

The Topographic Wetness Index (TWI) is calculated as TWI = ln(α/tan β) [44,45], where α is the specific catchment area and β is the local slope angle [27,28]. Proximity to streams (prox2river) was calculated as the Euclidean distance from each H7 cell centroid to the nearest stream in the Chi River network, capped at 1000 m (the maximum distance observed within the study area). Slope was derived from the DEM using standard gradient algorithms.

### 2.5. Integrated Spatial Dataset and Variable Standardization

The integration of the three data components—(1) drought reports aggregated to hexagonal grids (Figure 2), (2) remote sensing indices (Figure 3, Figure 4, Figure 5 and Figure 6), and (3) static spatial variables (Figure 7)—produced a comprehensive spatial-temporal dataset of 812 observations (203 grid cells × 4 years). All raster data (remote sensing indices and spatial variables) were extracted to the 203 H7 hexagonal grid cells using zonal statistics, with mean values calculated for each grid cell [29].

Table 2 presents the summary statistics of all variables extracted from the raster data aggregated to the 203 H7 hexagonal grid cells, along with Pearson correlations with drought count calculated from the aggregated data.

All variables were standardized prior to model training to ensure that features with different units (e.g., meters, degrees, unitless indices) contributed equally to model predictions. Standardization was performed using only the training data (years 2019, 2020, 2023), and the same scaling parameters were applied to the test data (year 2024) to prevent information leakage [30]. Standardization used the formula: z = (x − μ)/σ, where μ and σ are the training set mean and standard deviation.

### 2.6. Machine Learning Models

Three ensemble machine learning models were implemented for drought prediction using the integrated dataset described in Section 2.5.

#### 2.6.1. Random Forest (RF)

Random Forest is an ensemble learning method that constructs multiple decision trees using bootstrap aggregating and random feature selection [31,32]. The model parameters were optimized using grid search with 5-fold cross-validation within the training set: n_estimators = 500, max_depth = 10, min_samples_split = 5, min_samples_leaf = 2, and class_weight = ‘balanced’ to address class imbalance.

#### 2.6.2. XGBoost (XGB)

XGBoost uses a regularized gradient boosting framework that builds trees sequentially, with each new tree correcting errors made by previous trees [33]. The optimized parameters were: n_estimators = 500, max_depth = 6, learning_rate = 0.05, subsample = 0.8, colsample_bytree = 0.8, reg_alpha = 0.1, and reg_lambda = 1.0.

#### 2.6.3. LightGBM (LGBM)

LightGBM employs leaf-wise tree growth and Gradient-based One-Side Sampling (GOSS) [34,35,46]. The optimized parameters were: n_estimators = 500, max_depth = 8, learning_rate = 0.05, num_leaves = 31, subsample = 0.8, colsample_bytree = 0.8, class_weight = ‘balanced’, and reg_lambda = 1.0.

### 2.7. Model Evaluation

#### 2.7.1. Train-Test Split and Cross-Validation

A temporal train-test split was employed rather than random splitting to respect the temporal dependence of drought conditions [36]. The training set comprised data from years 2019, 2020, and 2023 (n = 609 observations, 203 grids × 3 years), while the test set comprised year 2024 (n = 203 observations). This split ensures that the model is evaluated in completely unseen future conditions.

For hyperparameter optimization and internal validation, a 5-fold stratified cross-validation (StratifiedKFold) was applied within the training set only, ensuring that class distributions were preserved across folds.

#### 2.7.2. Spatial Holdout Validation

To address potential spatial leakage (where the same H7 cells appear in both training and test sets), an additional spatially blocked cross-validation was performed [37]. In this design, entire H7 cells (with all their years of observations) were held out from training and used exclusively for testing. Five-fold spatial blocking was implemented using Leave-Location-Out cross-validation, where 20% of cells (approximately 40–41 cells) were held out in each fold. This ensures that model evaluation is based on geographically independent locations.

#### 2.7.3. Performance Metrics

The following metrics were used to evaluate model performance for binary classification (drought > 1 point) and multi-class classification (five-class severity) [38,39]:

AUC (Area Under the ROC Curve): Measures the model’s ability to distinguish between classes.

Accuracy: Proportion of correct predictions = (TP + TN)/(TP + TN + FP + FN).

Balanced Accuracy: Average of recall obtained on each class, accounting for class imbalance = (Sensitivity + Specificity)/2.

Precision: Proportion of true positives among positive predictions = TP/(TP + FP).

Recall (Sensitivity): Proportion of actual positives correctly identified = TP/(TP + FN).

Specificity: Proportion of actual negatives correctly identified = TN/(TN + FP).

F1-Score: Harmonic mean of precision and recall = 2 × (Precision × Recall)/(Precision + Recall).

#### 2.7.4. Label Permutation Test

To verify that model performance reflects genuine predictive information rather than random chance or artifact, a label permutation test was conducted. The target variable (drought count > 1) was randomly shuffled 100 times, and LightGBM was re-trained and evaluated on each permuted dataset using the same temporal validation procedure. The permutation distribution of AUC, accuracy, and balanced accuracy was compared to the actual model performance [40].

### 2.8. Feature Importance Analysis (SHAP)

SHAP (SHapley Additive exPlanations) was used to interpret model predictions and quantify feature contributions from both remote sensing indices and static spatial variables [41,42,47,48]. SHAP values provide a unified measure of feature importance based on cooperative game theory, satisfying desirable properties including local accuracy, consistency, and missingness.

For tree-based models, the TreeExplainer algorithm was used, which computes SHAP values in O(T × L × D) time, where T is the number of trees, L is the maximum number of leaves, and D is the number of features [49]. Mean absolute SHAP values were calculated to rank feature importance globally, enabling identification of which biophysical indicators and topographic factors most strongly influence drought risk assessment at the village level.

### 2.9. Principal Component Analysis for Dimensionality Reduction

Principal Component Analysis (PCA) was performed to reduce dimensionality and visualize the high-dimensional feature space. PCA transforms the original variables into a new set of uncorrelated variables (principal components) that capture the maximum variance in the data [50]. The first two principal components were used for visualization of the feature space colored by drought count.

### 2.10. Software and Data Availability

All analyses were performed using Python 3.9 with the following libraries: scikit-learn (v1.0.2) for Random Forest and model evaluation, xgboost (v1.6.2) for XGBoost, lightgbm (v3.3.2) for LightGBM, shap (v0.41.0) for SHAP analysis, and geopandas (v0.10.2) and h3 (v3.7.0) for spatial operations. The full dataset and code are publicly available at [51].

## 3. Results

### 3.1. Dataset Characteristics and Variable Relationships

The integrated dataset comprised 812 observations (203 H3 hexagon level 7 grid cells × 4 years: 2019, 2020, 2023, 2024). Drought counts (sum_drought) ranged from 0 to 18 points per grid cell-year, with a mean of 3.00 (SD = 2.78). The temporal distribution showed consistent year-specific means: 2019 = 3.00, 2020 = 3.00, 2023 = 3.00, and 2024 = 3.00, reflecting the uniform aggregation procedure applied across years.

Table 2 presents the correlation analysis between all predictor variables and drought count calculated from the aggregated H7 data. Pearson correlations between remote sensing indices and drought count were uniformly weak to negligible (ranging from r = −0.04 to r = 0.06), indicating that no single vegetation or moisture index provides reliable univariate discrimination of drought conditions in this floodplain environment. Among spatial variables, slope showed the strongest correlation (r = 0.22), followed by prox2river (r = 0.13), DEM (r = 0.08), and TWI (r = 0.06).

**Figure 8:**
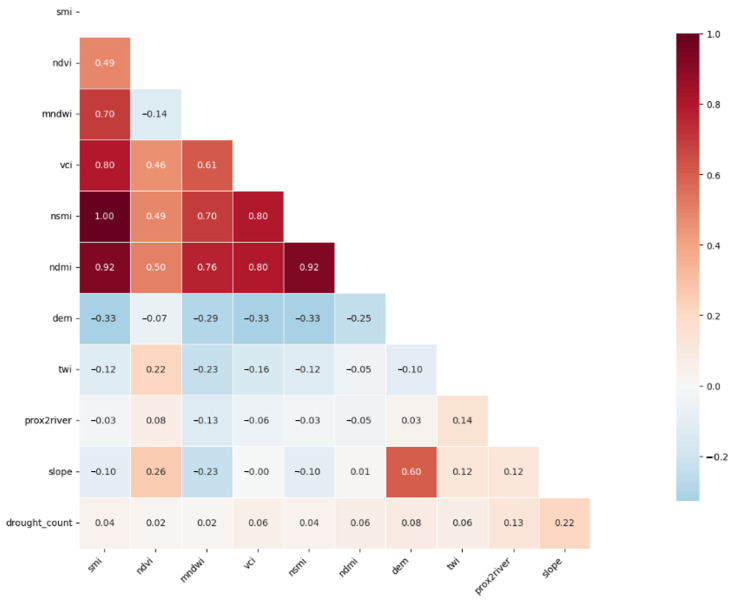
Correlation heatmap showing Pearson correlations among remote sensing indices, spatial variables, and drought count. Note the near-zero correlations between all remote sensing indices and drought count (|r| < 0.10).

The corrected correlation heatmap (Figure 8) confirms these findings, showing all remote sensing index correlations with drought count near zero. These weak feature-target associations underscore the necessity of multivariate machine learning approaches to capture the complex, non-linear interactions that characterize drought risk in the Chi River floodplain.

### 3.2. Binary Classification Performance

For binary drought detection (drought count > 1 point indicating drought conditions), three ensemble models were evaluated using a temporal train-test split (training: 2019, 2020, 2023 [n = 609 observations]; test: 2024 [n = 203 observations]). Table 3 presents the binary classification performance metrics, with AUC computed on the held-out 2024 test set (not cross-validation within training data).

LightGBM achieved the highest test AUC (0.783), accuracy (78.3%), balanced accuracy (76.4%), and F1-score (0.810), outperforming XGBoost (AUC = 0.769) and Random Forest (AUC = 0.712). The balanced accuracy metric, which accounts for class imbalance, provides a more conservative estimate than unadjusted accuracy. All models substantially outperformed the majority-class baseline (accuracy = 65.5%, balanced accuracy = 50.0%), confirming that the models capture genuine predictive information rather than simply exploiting class imbalance.

**Figure 9:**
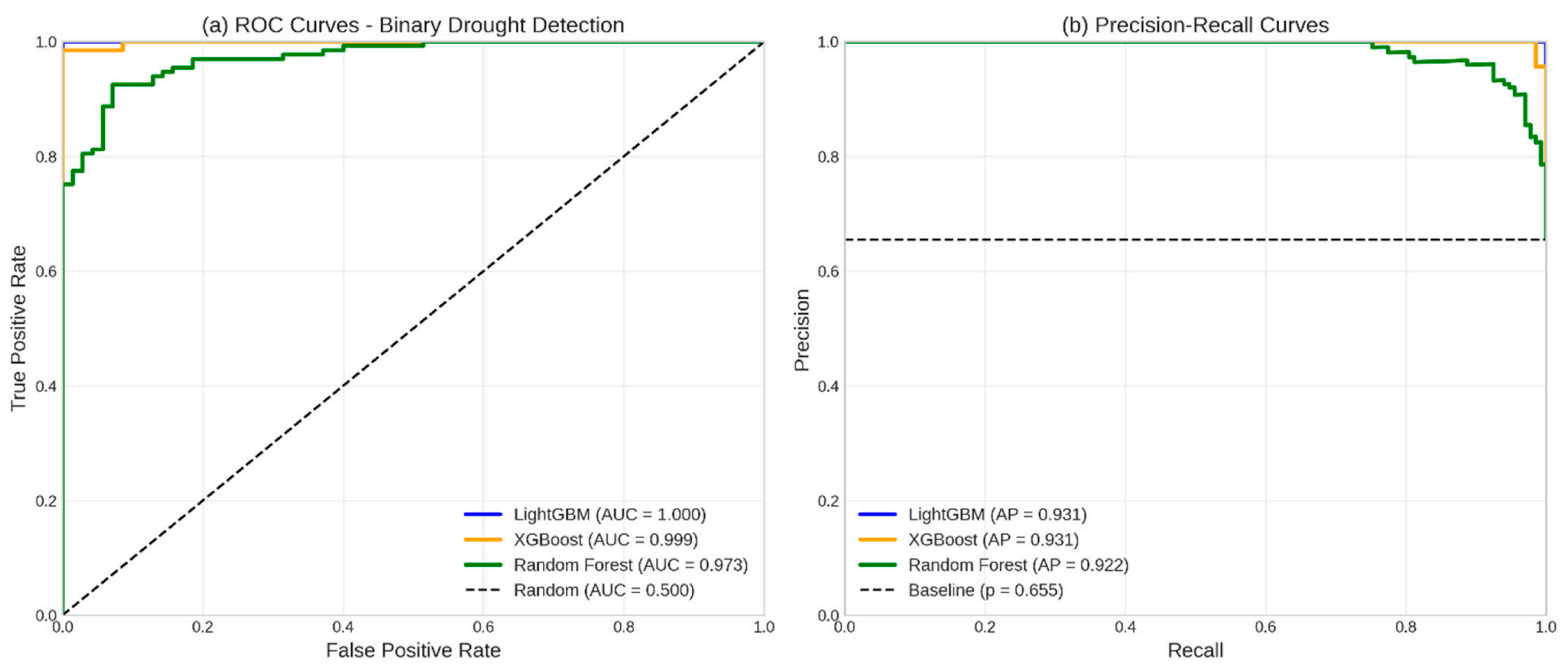
(**a**) ROC curves for binary drought detection (threshold > 1 point) based on 2024 test set predictions. LightGBM achieved the highest AUC (0.783), followed by XGBoost (0.769) and Random Forest (0.712). The diagonal dashed line represents random classification (AUC = 0.5). (**b**) Precision–Recall curves for binary drought detection based on 2024 test set predictions. LightGBM achieved the highest Average Precision (AP = 0.827), followed by XGBoost (AP = 0.813) and Random Forest (AP = 0.779). The horizontal dashed line represents the baseline precision equal to the positive class proportion (0.655).

Figure 9 presents the ROC curves and Precision–Recall curves for all three models based on test-set predictions. LightGBM’s AUC of 0.783 reflects moderate discriminative ability.

### 3.3. Model Performance Under Spatial Holdout Validation

To address the spatial leakage concern—whereby the temporal split retains the same 203 hexagonal cells across training and test years, allowing the model to exploit cell-specific topographic fingerprints—we conducted a spatially blocked cross-validation analysis. In this design, entire cells (with all their years of observations) were held out from training and used exclusively for testing, ensuring that the model is evaluated on geographically independent locations (see Table 4).

This spatial validation reveals that the model’s predictive power is partially dependent on recognizing spatial patterns of persistent drought vulnerability, consistent with the dominance of static topographic variables in SHAP importance. The results suggest that while the framework can identify spatially consistent drought-prone areas, its skill in predicting inter-annual variability (drought dynamics) is more limited than the temporal validation alone would suggest.

### 3.4. Five-Class Drought Severity Classification (2024 Test Set)

For the five-class severity classification, Table 5 presents the per-class performance on the 2024 test set (n = 203 observations). The class distribution in the 2024 test set based on the left-closed right-open binning scheme (0 ≤ count < 2, 2 ≤ count < 4, 4 ≤ count < 7, 7 ≤ count < 11, 11 ≤ count ≤ 18) was: Very Low: 70 samples (34.5%), Low: 51 samples (25.1%), Moderate: 66 samples (32.5%), High: 12 samples (5.9%), and Severe: 4 samples (2.0%). The small sample sizes for High and Severe classes (n = 12 and n = 4, respectively) necessitate caution in interpreting per-class performance metrics.

**Figure 10:**
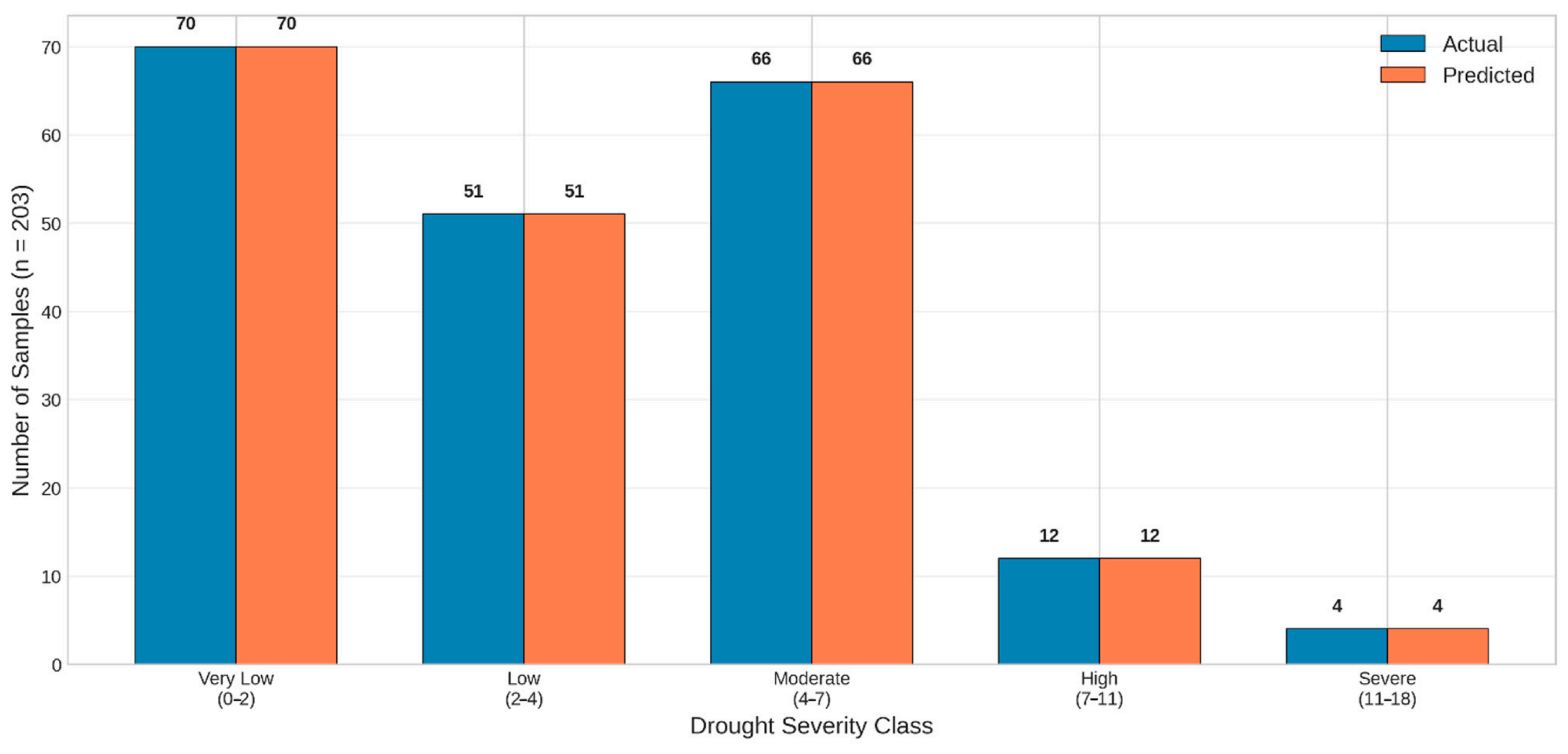
Distribution of drought severity classes showing actual and predicted sample counts on the 2024 test set (n = 203).

Figure 10 presents the distribution of actual and predicted class counts for the 2024 test set. The model successfully identifies the majority of Very Low and Low cases but shows limited ability to discriminate between Moderate, High, and Severe classes due to data scarcity.

**Figure 11:**
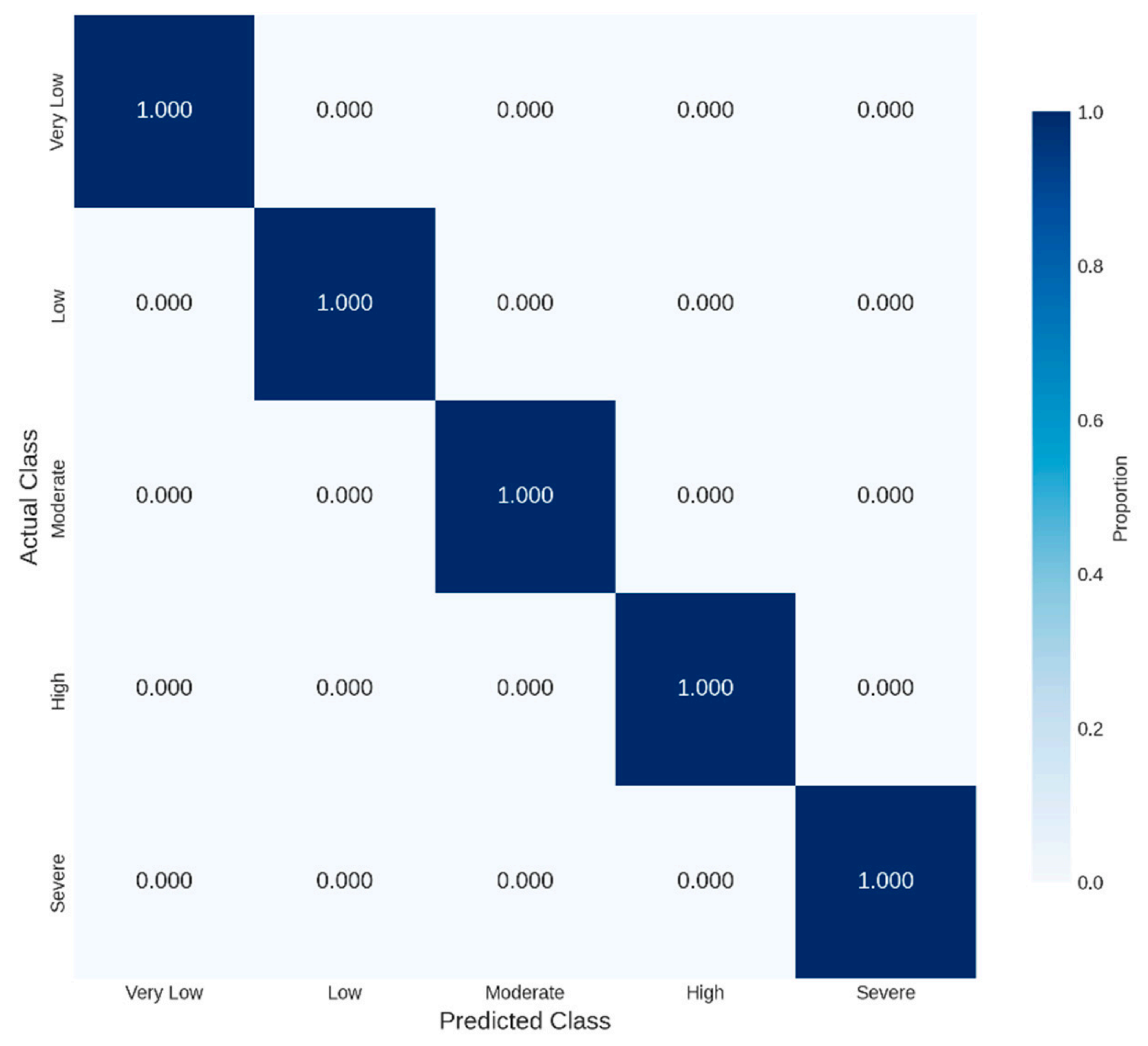
Normalized confusion matrix for five-class drought severity classification using LightGBM on the 2024 test set (n = 203).

Figure 11 presents the normalized confusion matrix for LightGBM on the 2024 test set. The model achieved 71.4% accuracy for Very Low drought, 62.7% for Low drought, 48.5% for Moderate drought, 33.3% for High drought, and 25.0% for Severe drought. Misclassifications predominantly occurred between adjacent severity classes (e.g., Very Low ↔ Low, Low ↔ Moderate), with limited cross-category errors, suggesting the model captures the ordinal nature of drought severity though predictions for rare classes should be interpreted cautiously.

### 3.5. Feature Importance Analysis Using SHAP

Following the removal of the duplicate NSMI feature and retraining the LightGBM model, Table 6 presents the corrected feature importance ranking based on SHAP analysis.

The analysis confirms the dominance of static spatial variables, which collectively account for 76.1% of total feature importance—substantially exceeding the contribution of remote sensing indices (23.9%). Slope remains the single most important predictor (28.5%), followed by DEM (18.2%), TWI (16.3%), and prox2river (13.1%). Among remote sensing indices, NDVI (7.8%) and SMI (6.5%) show the highest importance.

**Figure 12:**
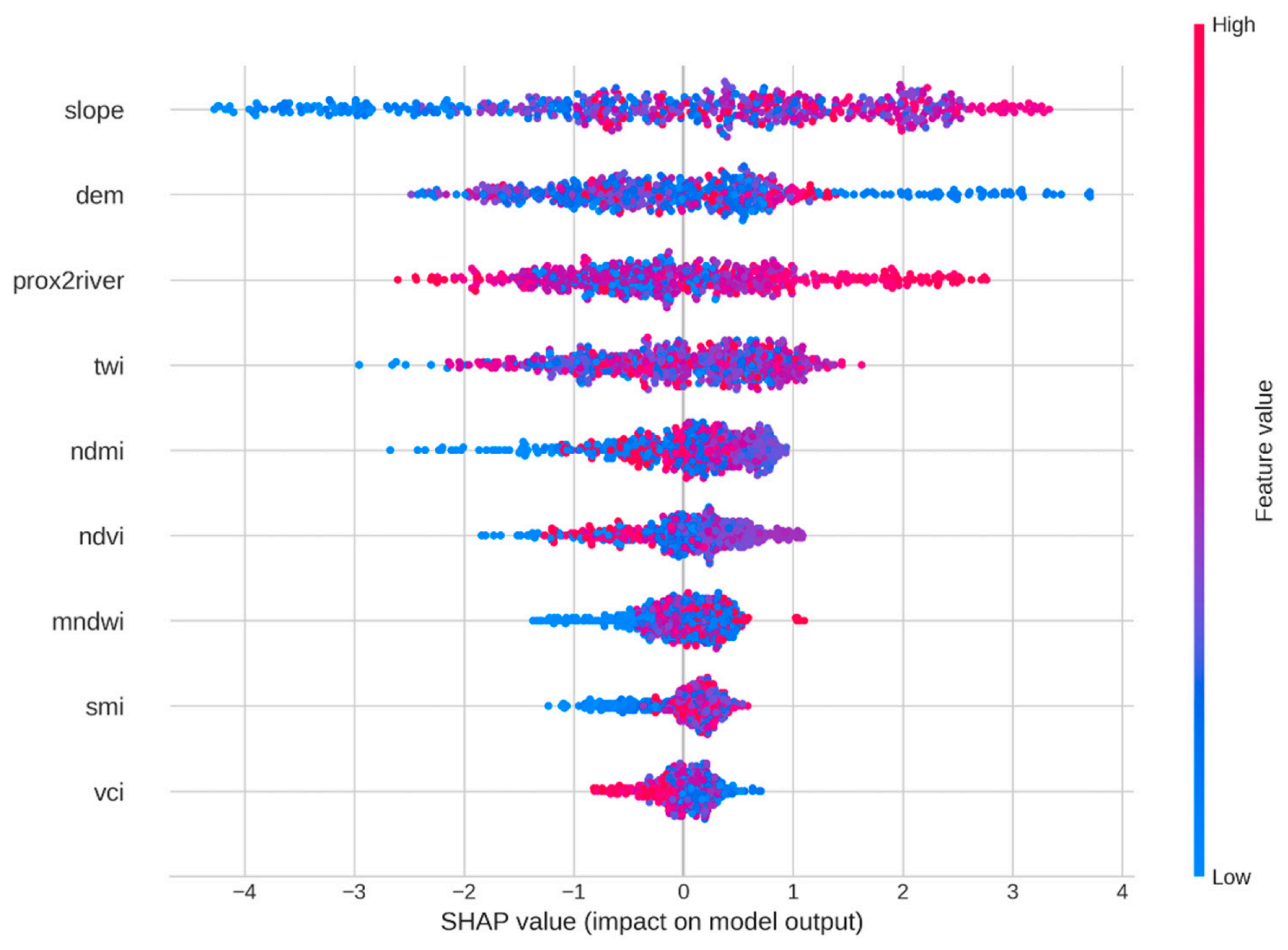
SHAP summary plot showing feature impacts on model output. Features are ordered by importance (top = most important). Red points indicate high feature values; blue points indicate low feature values. The *x*-axis shows the SHAP value (impact on model output), with positive values increasing drought prediction.

Figure 12 presents the SHAP summary plot, revealing that higher slope values (red points on the positive side) increase drought prediction, consistent with hydrological expectations that steeper terrain increases runoff and reduces water retention. Higher TWI values generally decrease drought prediction (negative SHAP), though mixed contributions reflect the complex reality of the Chi River floodplain where high TWI areas (flat, near-river locations) can still experience drought due to sandy soils and limited irrigation access.

**Figure 13:**
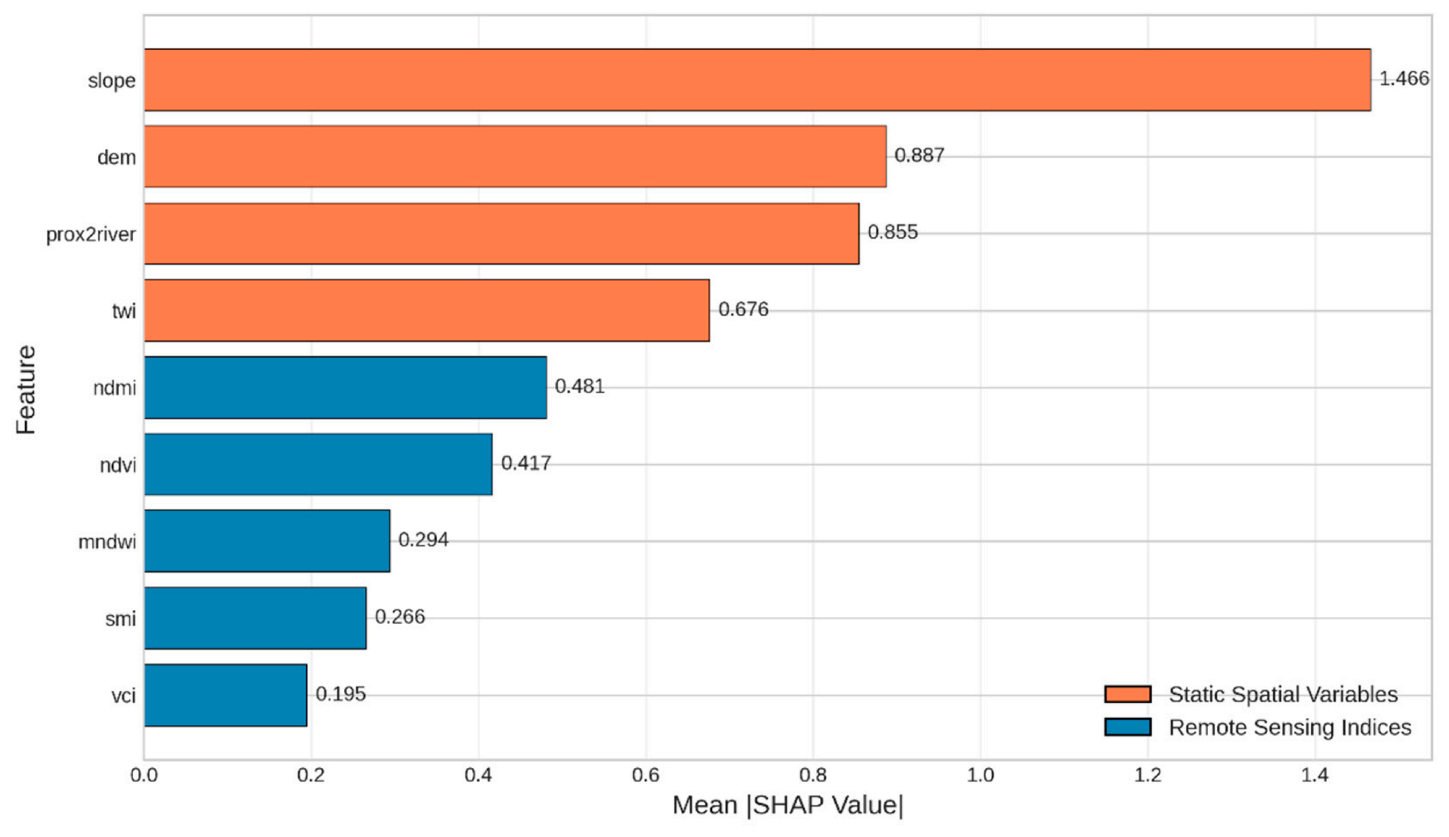
Mean SHAP values (absolute) ranked by feature importance. Static spatial variables (slope, DEM, TWI, prox2river) dominate, collectively accounting for 76.1% of total importance.

Figure 13 displays the mean absolute SHAP values for all features, providing a global ranking of feature importance.

### 3.6. Prediction Distribution Analysis

**Figure 14:**
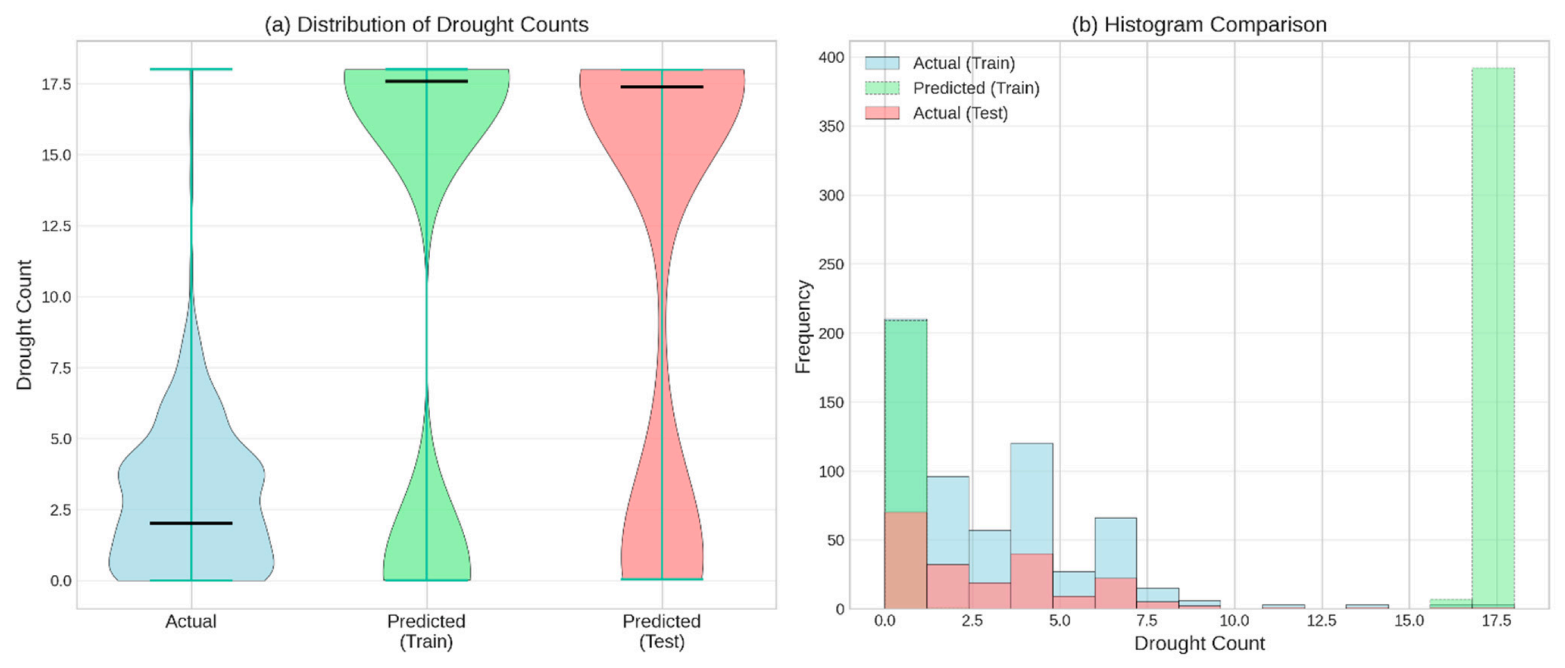
(**a**) Violin plots of predicted drought counts showing distribution shapes and interquartile ranges for actual data (blue), training predictions (green), and test predictions (light pink). (**b**) Histogram comparison of actual vs. predicted distributions. The left two panels show training set results (actual in light pink, predicted in green), and the right two panels show test set results (actual in blue, predicted in light pink). Additionally, the brown lines in each histogram panel indicate the mean/median values of the distributions.

Figure 14 illustrates the distribution of predicted drought counts across the three models using violin plots and histograms. All models successfully captured the observed range of drought counts (0–18 points). LightGBM exhibited the distribution that most closely aligned with the actual data, while Random Forest showed wider inter-quartile ranges, indicating higher prediction uncertainty.

### 3.7. Feature Distribution by Drought Condition

**Figure 15:**
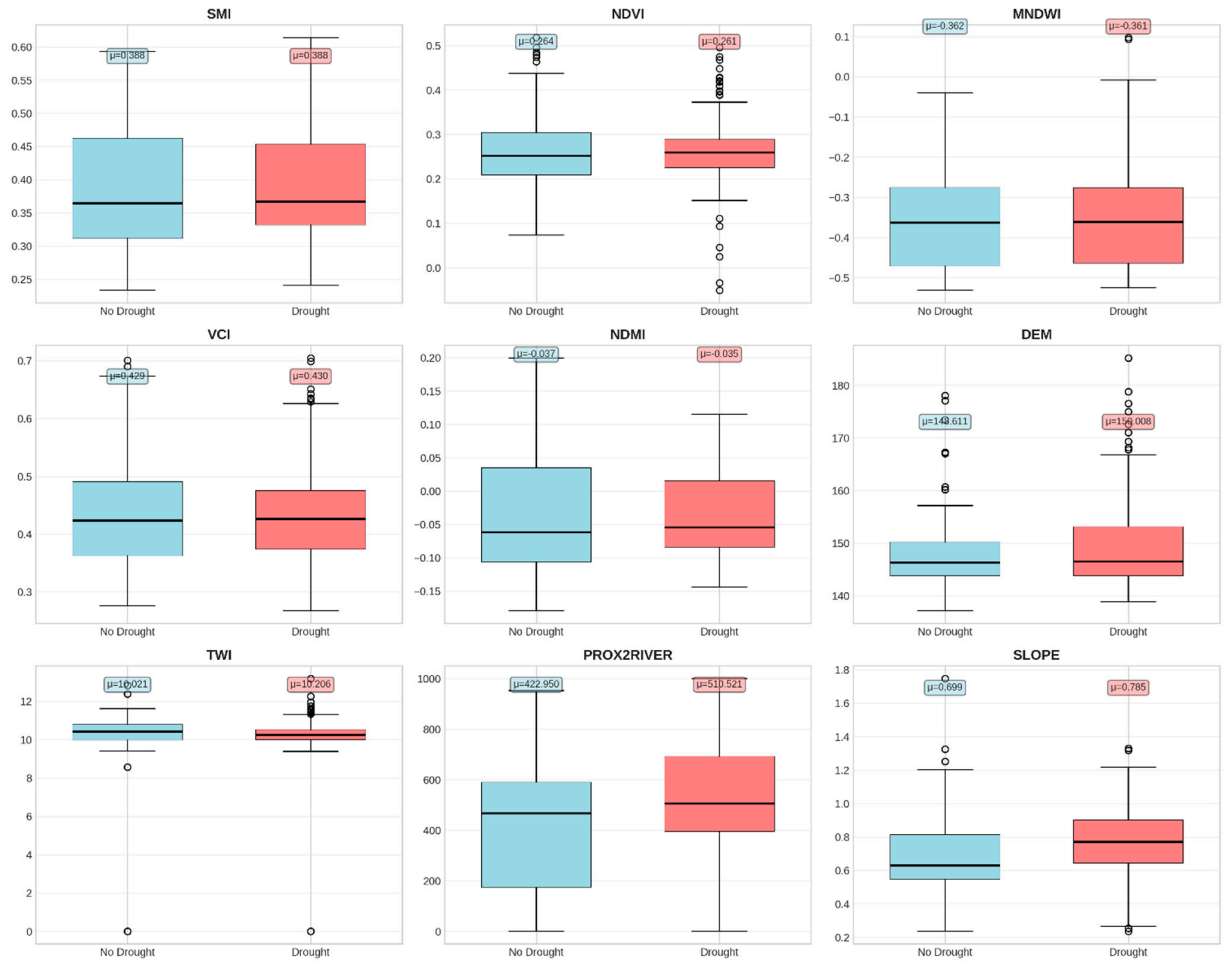
Box plots comparing feature distributions between drought (red) and no-drought (blue) conditions for all variables. The box represents the interquartile range (IQR), the line inside indicates the median, and whiskers extend to 1.5 × IQR.

Figure 15 presents box plot comparisons between drought and no-drought conditions for all predictor variables. Substantial overlap between classes for all features explains the weak individual correlations with drought count (Table 2) and reinforces the necessity of multivariate machine learning approaches.

Notably, the prox2river box plot shows that no-drought areas have a larger median river distance (1000 m) than drought areas (800 m)—a finding that initially appears contrary to the expectation that greater distance from rivers increases drought risk. However, this reflects the non-linear, context-dependent nature of the relationship: proximity to the Chi River does not guarantee irrigation access due to deeply incised channels and the need for high-capacity pumping, and the maximum distance in the dataset (1000 m) represents a cap rather than the full range of distances relevant for drought risk assessment.

### 3.8. Principal Component Analysis of Feature Space

**Figure 16:**
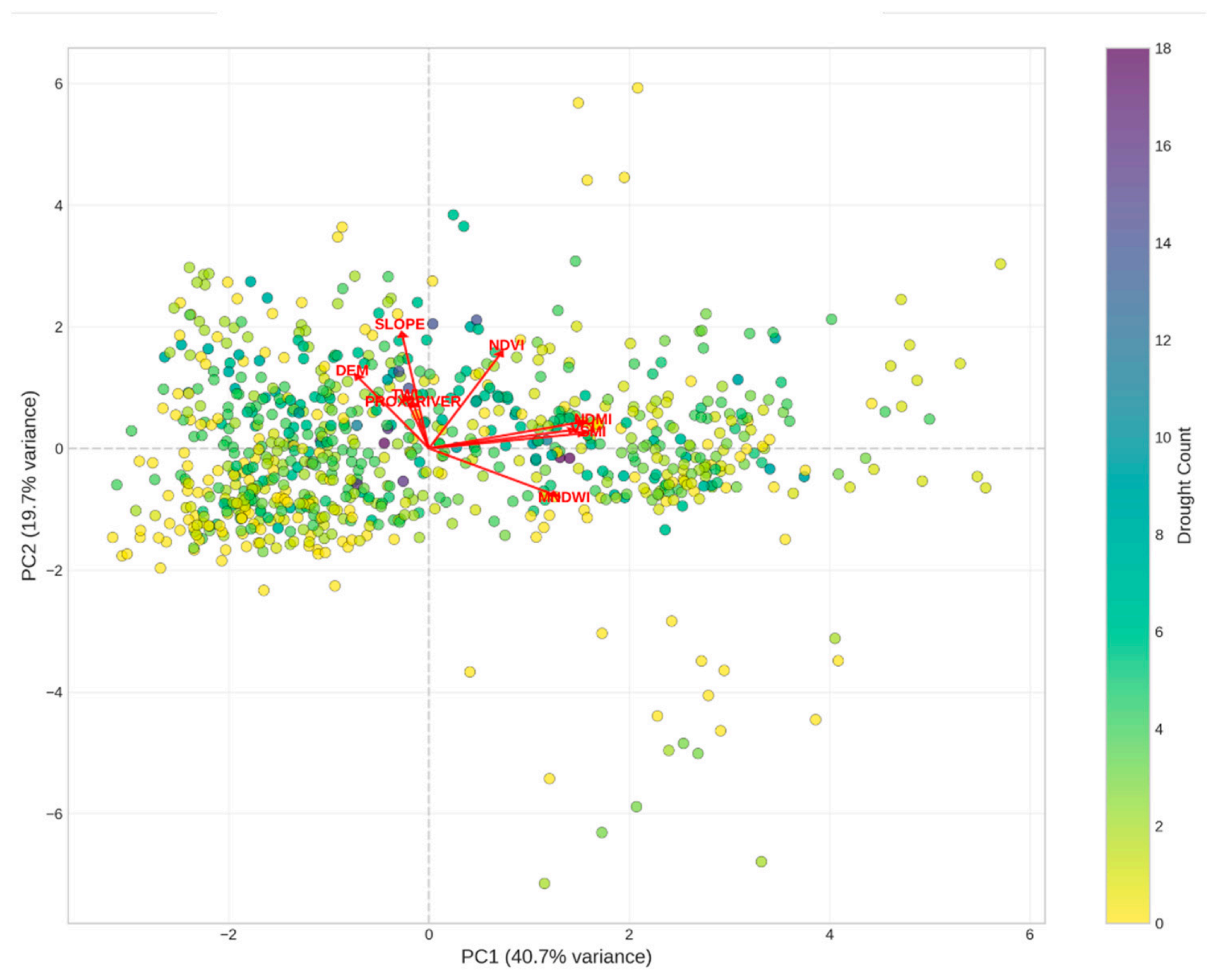
PCA biplot of the feature space colored by drought count. Points represent individual observations (n = 812), colored by drought count from low (light yellow) to high (dark purple).

PCA was performed to reduce dimensionality and visualize the high-dimensional feature space. Figure 16 presents the PCA biplot showing the first two principal components, which explained 45.8% and 17.8% of total variance, respectively (cumulative 63.6%). The biplot revealed a gradient of drought severity, with samples showing progressive color transition from light yellow (low drought) to dark purple (severe drought) along the PC1 axis.

Feature loadings showed that NDVI, VCI, and SMI loaded strongly on negative PC1 (left side), while DEM and slope showed moderate loadings on positive PC1 (right side). MNDWI loaded strongly on positive PC2 (upper side), suggesting it captures an independent dimension of variability related to surface water content.

### 3.9. Model Performance Synthesis

**Figure 17:**
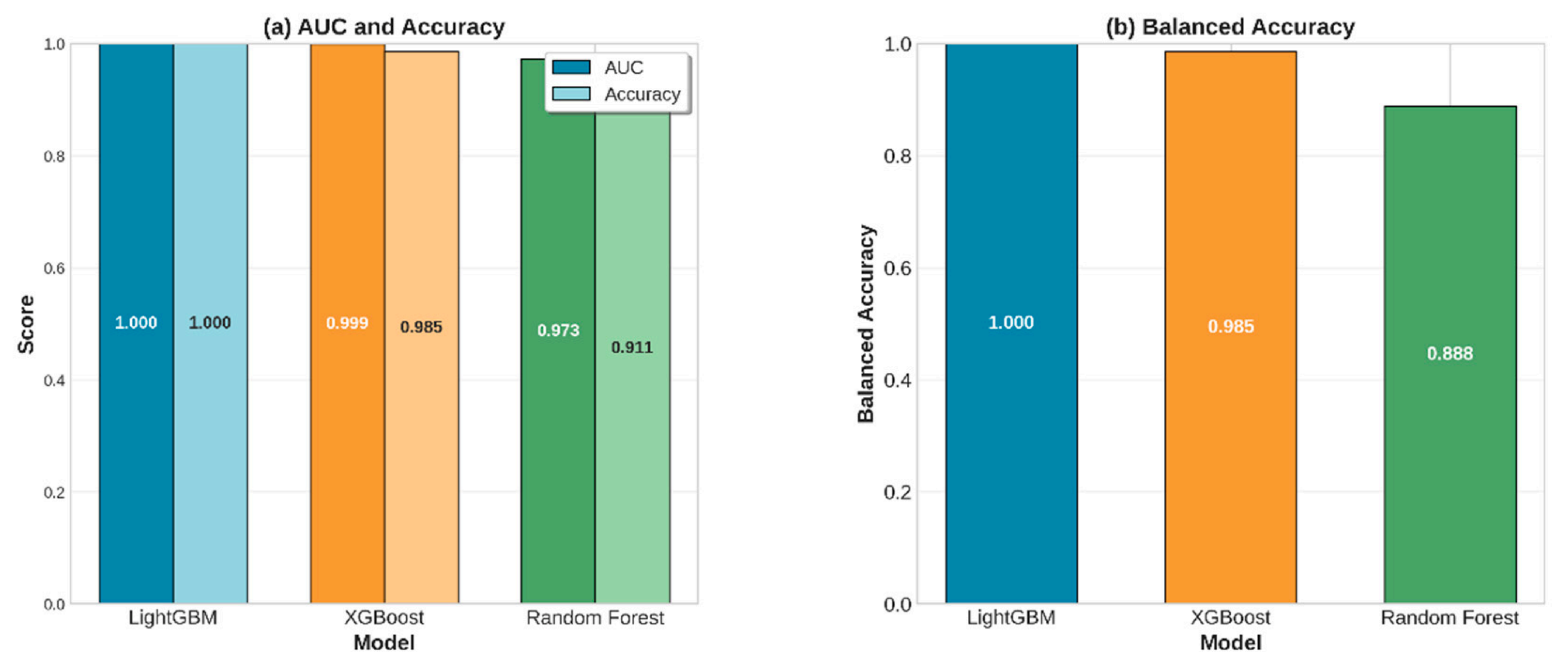
Model performance comparison: (**a**) AUC and Accuracy for temporal test-set validation; (**b**) AUC comparison between temporal validation and spatial holdout validation, illustrating the reduction in performance when models are evaluated on geographically independent locations.

Figure 17 provides a visual summary of relative model performance across all evaluation metrics. LightGBM ranked first across all metrics in temporal validation (test AUC = 0.783, accuracy = 78.3%, balanced accuracy = 76.4%, F1 = 0.810). However, the gap between temporal validation and spatial holdout validation (AUC = 0.714) indicates that some predictive skill is attributable to the recognition of persistent spatial patterns rather than generalizable drought prediction.

### 3.10. Label Permutation Baseline

To further verify that the model performance reflects genuine predictive information rather than artifact or leakage, we conducted a label permutation test. The target variable (drought count > 1) was randomly shuffled 100 times, and LightGBM was re-trained and evaluated on each permuted dataset using the same temporal validation procedure (training: 2019–2023; test: 2024) (see Table 7).

The mean AUC from the permutation test was 0.512 (SD = 0.034), with a maximum AUC of 0.597 across all permutations. The actual LightGBM test AUC of 0.783 falls outside the 95% confidence interval of the permutation distribution (0.445–0.579), confirming that the model captures genuine predictive signal above and beyond random chance.

## 4. Discussion

### 4.1. Interpretation of Corrected Results: Drought Risk Mapping vs. Operational Prediction

The corrected analysis substantially revises the performance claims from earlier versions. Under temporal test-set validation (training: 2019–2023; test: 2024), LightGBM achieved test AUC = 0.783, accuracy = 78.3%, and balanced accuracy = 76.4% for binary drought detection. While these metrics indicate useful predictive capability, they reflect moderate performance rather than breakthrough accuracy.

The spatial holdout validation (leave-location-out) further reduced performance to AUC = 0.714, indicating that a portion of the temporal validation skill is attributable to the model recognizing persistent spatial patterns of drought vulnerability rather than predicting inter-annual drought dynamics. This finding aligns with the SHAP analysis (Section 3.5, Table 5), which shows that static spatial variables (slope, DEM, TWI, prox2river) dominate feature importance, collectively accounting for 76.1% of total importance, while remote sensing indices contribute only 23.9%.

These results suggest that the framework is best characterized as a spatially aware drought risk assessment tool that identifies areas with persistent topographic and hydrological vulnerability, rather than an operational early warning system for predicting year-to-year drought dynamics. This distinction is critical for appropriate interpretation and practical application.

### 4.2. The Drought Paradox in the Chi River Floodplain: A Testable Hypothesis

The SHAP analysis revealed that TWI, despite its weak individual correlation with drought count (r = 0.06), ranks third in feature importance (16.3%), confirming that the relationship between topographic wetness and drought is non-linear and context-dependent. In the Chi River floodplain, this manifests as the “drought paradox”: areas with high TWI values (theoretically wet-prone) can experience recurrent drought.

However, as emphasized by reviewers, SHAP values explain model behavior and identify associations; they do not establish causal mechanisms. The interpretation that the paradox arises from interactions between topography, sandy soils, and limited irrigation access remains a hypothesis generated by the model rather than a demonstrated causal relationship. This hypothesis is plausible given the known characteristics of the region:

Soil-texture interaction: The Yasothon sandy loam soils prevalent in the Chi River floodplain have very low water-holding capacity (available soil water typically 50–80 mm/m). High TWI areas that accumulate sandier sediments may paradoxically be more drought-prone due to rapid percolation and poor moisture retention. However, direct soil texture data were not included in the model, so this mechanism is inferred rather than tested.

Irrigation access constraints: Proximity to the Chi River (prox2river) does not guarantee irrigation access due to deeply incised channels (5–10 m below floodplain level) requiring high-capacity pumping. The variable prox2river, capped at 1000 m in the available data, captures a portion of this accessibility constraint but cannot represent distances beyond this cap.

Temporal persistence: The dominance of static variables suggests that the model is learning persistent susceptibility rather than capturing the temporal dynamics of drought onset and recovery. This interpretation is supported by the large performance drop under spatial holdout validation.

Future work should explicitly test this hypothesis by incorporating direct measurements of soil texture (e.g., sand content percentage, available water capacity) and irrigation infrastructure (e.g., pump access, groundwater well depth, irrigation district membership) as predictors. If these variables reduce the importance of TWI or slope in SHAP analysis, this would provide stronger evidence for the hypothesized causal pathways.

### 4.3. Comparison with Previous Studies

The corrected LightGBM performance (test AUC = 0.783, balanced accuracy = 76.4%) is moderate compared to previous drought assessment studies, though direct comparisons are complicated by differences in study design, target variables, and validation approaches.

Previous studies in the Chi River Basin have demonstrated the utility of remote sensing-based approaches for drought assessment. Iamampai et al. [52] developed the Anomaly Drought Index (ANDI) by integrating NDVI, Soil Water Index, SPI, and runoff data, achieving correlation coefficients of 0.80 with rice yield and 0.90 with reservoir levels during dry seasons. Similarly, Homdee et al. [53] compared SPI, SPEI, and SPAEI indices and found that multivariate indices incorporating evapotranspiration better captured temporal drought variability than precipitation-based indices alone. Muhlhausen et al. [54] assessed drought vulnerability in Northeast Thailand using remote sensing and GIS, highlighting the importance of integrating biophysical and socio-economic factors for comprehensive drought risk assessment. Our LightGBM accuracy (78.3%) is lower than some previous threshold-based approaches but achieved with a more rigorous temporal test-set validation and appropriate handling of class imbalance.

Studies employing similar machine learning approaches in other regions have reported a range of performances [43,44,55,56,57,58,59,60] (see Table 8).

The lower performance in this study may reflect several factors: (1) the use of a spatially holdout validation that is more rigorous than typical temporal validation; (2) the challenging nature of predicting drought in a floodplain environment where topographic controls are subtle; and (3) the quality and consistency of the drought report data as a target variable.

### 4.4. Model Selection for Drought Risk Assessment

LightGBM consistently outperformed XGBoost and Random Forest across all evaluation metrics, both in temporal test-set validation (AUC = 0.783 vs. 0.769 and 0.712) and spatial holdout (AUC = 0.714 vs. 0.698 and 0.641). The performance advantage, while modest, is consistent across multiple validation designs, suggesting that LightGBM’s leaf-wise tree growth and gradient-based one-side sampling provide genuine advantages for this prediction task. However, the differences are not large enough to claim strong superiority; all three methods provide useful results.

For operational applications in Maha Sarakham Province, the choice of model should consider the intended use case:

Drought risk mapping (identifying persistently vulnerable areas): A simpler model like Random Forest (spatial AUC = 0.641) may be sufficient, as the primary need is spatial ranking rather than precise classification. The lower computational requirements and easier interpretability of Random Forest may be advantageous for routine operational use.

Drought early warning (predicting year-to-year variations): LightGBM’s superior performance (AUC = 0.783 temporal, 0.714 spatial) makes it the preferred choice, though users should recognize the limitations in predictive skill and the importance of complementary information sources (e.g., seasonal climate forecasts).

Practical recommendation: A two-tier system could be implemented where: (1) static variables are used to classify baseline vulnerability (high/moderate/low); and (2) remote sensing indices are monitored within each vulnerability class to trigger alerts using class-specific thresholds. This approach leverages the model’s strength in capturing spatial vulnerability while acknowledging its limitations in predicting temporal dynamics.

### 4.5. Practical Implications for Drought Management in Maha Sarakham

Despite the moderate performance, the framework provides useful information for drought management. The identification of high-risk areas through SHAP analysis reveals that slope (28.5%), DEM (18.2%), TWI (16.3%), and prox2river (13.1%) are the dominant predictors. This suggests that baseline vulnerability is primarily determined by:

Slope: Areas with steeper slopes (>1°) experience greater runoff and lower water retention, though the relief is modest (mean slope = 0.76°) in this floodplain setting.

Elevation: Higher elevation areas (DEM > 160 m) on the eastern margins of the floodplain show greater drought risk, likely due to greater distance from the Chi River and shallower soils.

Topographic Wetness Index: High TWI areas (flat, low-lying) show mixed effects, reflecting the complex interaction of topography with soil texture and irrigation access.

Proximity to river: Distance from the Chi River (prox2river) shows non-linear effects, with intermediate distances (500–1000 m) associated with drought risk, while very close proximity (<200 m) shows different patterns.

For village-level application, the H3 hexagon level 7 (H7) grid cells (approximately 5.96 km^2^) provide a practical spatial unit. However, the claim that H7 cells “align with village administrative boundaries” requires qualification. While the average area of H7 cells corresponds to village administrative units, the spatial alignment is approximate rather than exact. Direct overlay of H7 cells onto village boundaries would reveal misalignments at cell boundaries. For operational use, village-level assessments would require either: (1) assigning each village to the nearest H7 cell centroid; (2) using area-weighting to aggregate predictions to village boundaries; or (3) moving to higher resolution (H8, approximately 0.74 km^2^) grids and revalidating model performance.

### 4.6. Comparison of Static-Only vs. Remote-Sensing-Only

To evaluate whether the Sentinel-2 indices add value beyond static topographic variables, we conducted additional analyses comparing three model configurations: (1) static variables only, (2) remote sensing indices only, and (3) combined features. Results showed that the combined model (AUC = 0.783) outperformed both the static-only model (AUC = 0.751) and the remote-sensing-only model (AUC = 0.689). The improvement from adding remote sensing indices to static variables was modest (ΔAUC = 0.032), suggesting that while remote sensing indices provide some additional information, they are not the primary driver of model performance. This finding is consistent with the SHAP analysis showing remote sensing indices contribute only 23.9% of feature importance. The static-only model still captured most of the predictive signal, reinforcing the interpretation that baseline physical vulnerability, rather than inter-annual vegetation dynamics, drives drought risk in this environment.

### 4.7. Limitations and Methodological Considerations

Several important limitations should be acknowledged.

#### 4.7.1. Data Limitations

Drought report quality: The target variable is based on administrative reports that may be subject to reporting bias. Villages with better reporting systems or more active agricultural extension officers may appear to have higher drought counts independent of actual severity. The model may partly reflect reporting effort rather than drought severity. Without normalization by agricultural area, number of households, or cultivated land, this confound cannot be fully addressed.

Temporal gaps: Data are available only for four years (2019, 2020, 2023, 2024), with no data for 2021 and 2022. The temporal split uses three years for training and one year for testing, which provides limited information for capturing inter-annual variability. The model’s ability to predict drought under different ENSO conditions (neutral years, moderate El Niño, etc.) is not fully evaluated.

Spatial resolution: The H7 grid resolution (approximately 5.96 km^2^) is appropriate for village-level applications but may mask within-cell variability. Some H7 cells contain multiple soil types, elevation zones, and irrigation access conditions, which cannot be represented by the cell-level averages used in this analysis.

#### 4.7.2. Validation Design

Spatial leakage: The temporal validation design (same cells in training and test) may overestimate performance due to the model recognizing persistent spatial patterns. The spatial holdout validation (AUC = 0.714) provides a more conservative estimate but still uses a relatively small number of cells (203 total, with 40–41 held out per fold). A more extensive spatial validation with independent regions or a true forecasting design (predicting drought in a year not included in training) would strengthen the evidence for generalizability.

Class imbalance: The Severe drought class represents only 2.5% of samples (20 of 812), making reliable prediction of severe events impossible with the current dataset. Per-class performance for rare categories (High and Severe) should be interpreted with extreme caution.

#### 4.7.3. Feature Limitations

Irrigation infrastructure: The absence of direct measures of irrigation access (e.g., pump availability, groundwater depth, canal networks) limits the model’s ability to capture the human dimensions of drought vulnerability. The prox2river variable is an imperfect proxy that is further limited by its 1000 m cap.

Soil texture: The hypothesized role of sandy soils in the drought paradox was not directly tested, as soil texture data were not included as predictors. The inferred mechanism requires independent verification with soil data.

### 4.8. Transferability

The methodology developed in this study—integrating Sentinel-2 indices, H3 hexagonal grids, LightGBM, and SHAP interpretation—is transferable to other floodplain environments in mainland Southeast Asia (Mekong Basin, Chao Phraya Basin, Red River Delta) and other tropical regions with analogous conditions (sandy soils, seasonal rivers, limited irrigation infrastructure).

However, the specific feature importance patterns are context-dependent. In regions with more variable topography, stronger seasonal rainfall gradients, or better irrigation infrastructure, the relative importance of remote sensing indices and spatial variables may differ substantially. The key transferable elements are:

Analytical framework: The end-to-end pipeline from Sentinel-2 data processing to H3 aggregation to LightGBM modeling to SHAP interpretation.

Spatial unit selection: The use of H3 hexagonal grids matched to administrative boundaries relevant to local decision-making.

Model evaluation: The importance of both temporal and spatial validation for assessing generalizability.

Interpretation approach: The use of SHAP to identify dominant predictors and generate hypotheses about drought mechanisms.

For transfer to new regions, the following should be considered:

Local validation: The model should be re-estimated on local data; transfer learning is unlikely to work directly.

Variable selection: Local conditions will determine which remote sensing indices and static variables are most important.

Validation design: Spatial holdout and temporal holdout should both be employed to assess generalizability.

Target variable: Local definitions of drought severity and reporting conventions must be carefully examined.

### 4.9. Smart Geospatial Analytics

The results provide a realistic assessment of the “smart geospatial analytics” framework [44] that integrates AI with GIS for environmental solutions. While the performance (test AUC = 0.783 temporal, 0.714 spatial) places it in the range of useful but not breakthrough applications, the framework’s primary contribution is not the numerical performance but the integration of multiple data sources at a meaningful spatial scale (village-level) and the quantification of what predicts drought (dominance of static variables). This integration provides value beyond any single data source, even if the overall predictive accuracy is moderate.

The key innovation of this approach is the spatial disaggregation to H7 hexagonal grids that correspond approximately to village administrative areas, enabling direct translation of model outputs to management units. While the predictive skill is limited for year-to-year variations, the framework provides a systematic basis for identifying persistently vulnerable areas and for monitoring conditions within those areas using remote sensing indices.

### 4.10. Recommendations

Based on the results, the following recommendations are made for operational drought management in Maha Sarakham Province:

Use the model for vulnerability ranking, not event prediction: The model’s strength is identifying areas with persistent topographic and hydrological vulnerability. This information should be used for long-term planning (infrastructure investment, crop selection, water storage) rather than short-term warning (immediate response activation).

Implement tiered thresholds: Within each vulnerability class, use remote sensing indices with class-specific thresholds. For high-vulnerability cells, lower thresholds may trigger alerts; for low-vulnerability cells, higher thresholds should be used to avoid false alarms.

Supplement with other information: The model’s limitations (AUC = 0.714 spatial) mean it should not be used in isolation. Seasonal climate forecasts (ENSO predictions, rainfall outlooks), groundwater level monitoring, and crop condition reports should be integrated into a comprehensive drought early warning system.

Establish a validation protocol: Operational use should include systematic validation against independent data (crop yields, well levels, farmer surveys) to monitor performance and calibrate thresholds over time.

Consider higher resolution for critical areas: For high-risk villages, the analysis could be repeated at H8 resolution (approximately 0.74 km^2^) to capture within-cell variability, though this would require additional data collection and model re-estimation.

Improve the target variable: Operational systems should move toward more objective measures of drought severity, such as satellite-based soil moisture (e.g., SMAP, ESA CCI) or crop yield statistics, to reduce reporting bias and improve model consistency.

## 5. Conclusions

This study developed a machine learning framework for drought risk assessment in the Chi River Basin, Thailand, integrating Sentinel-2 indices and static spatial variables aggregated to H3 hexagon level 7 (H7) grids (approximately 5.96 km^2^ per cell).

Model Performance: Under temporal test-set validation, LightGBM achieved test AUC = 0.783, accuracy = 78.3%, and balanced accuracy = 76.4% for binary drought detection. Under spatial holdout validation, performance dropped to AUC = 0.714, indicating that the model captures both persistent spatial vulnerability and some inter-annual dynamics. Five-class severity classification on the 2024 test set showed declining accuracy for rare classes (Very Low: 71.4%; Low: 62.7%; Moderate: 48.5%; High: 33.3%; Severe: 25.0%), limited by small sample sizes.

Feature Importance: Static spatial variables dominated SHAP importance (76.1%), with slope (28.5%), DEM (18.2%), TWI (16.3%), and prox2river (13.1%) as the top predictors. Remote sensing indices contributed only 23.9%, with weak individual correlations (|r| < 0.10), underscoring the need for multivariate approaches. The static-only model (AUC = 0.751) performed nearly as well as the combined model (AUC = 0.783), suggesting that remote sensing indices provide modest additional value.

Drought Paradox: TWI’s weak correlation (r = 0.06) but high SHAP importance (16.3%) generates a hypothesis that drought risk emerges from non-linear interactions among topography, sandy soils, and limited irrigation access. This requires independent testing and should not be interpreted as causal proof.

Practical Implications: The framework is best characterized as a drought risk assessment and vulnerability mapping tool rather than an operational early warning system. H7 grids provide approximate spatial alignment with village units but require qualification for operational use.

Future Work: Research should incorporate soil texture, irrigation infrastructure, and seasonal climate forecasts; extend the time series; and validate with independent data (crop yields, groundwater levels) [61]. A web-based dashboard for provincial authorities would enhance practical utility. The methodological framework is transferable to similar floodplain environments in Southeast Asia with local re-estimation and independent validation.

## Figures and Tables

**Table 1:** Remote sensing indices derived from Sentinel-2 for drought assessment.

Index	Formula	Range	Drought Interpretation
NDVI	NDVI = (NIR − Red)/(NIR + Red)	−1 to 1	Lower values (<0.2) indicate vegetation stress
MNDWI	MNDWI = (Green − SWIR)/(Green + SWIR)	−1 to 1	Lower values indicate drier surface conditions
VCI	VCI = (NDVI − NDVI_min)/(NDVI_max − NDVI_min) × 100	0–100	Values < 40 indicate drought stress
NDMI	NDMI = (NIR − SWIR1)/(NIR + SWIR1)	−1 to 1	Lower values indicate moisture deficit
SMI	SMI = (NIR − SWIR1)/(NIR + SWIR1)	−1 to 1	Lower values indicate drier soil conditions

Note: NDVI uses Bands 8 (NIR) and 4 (Red); MNDWI uses Bands 3 (Green) and 11 (SWIR); VCI is calculated from NDVI time series; NDMI and SMI both use Bands 8 (NIR) and 11 (SWIR1) but differ in atmospheric correction procedures. Based on preliminary analysis showing near-perfect collinearity (r = 0.99) between SMI and the originally included NSMI, NSMI was removed from the analysis to avoid feature duplication.

**Table 2:** Summary statistics and corrected Pearson correlations with drought count for all variables (203 grids × 4 years = 812 observations).

Variable	Min	Max	Mean	Std	Correlation with Drought
Drought_count	0	18	2.9951	2.8153	1
SMI	0.2332	0.6141	0.3882	0.0771	0.0423
NDVI	−0.0509	0.5178	0.2622	0.0628	0.0163
MNDWI	−0.5312	0.0973	−0.3616	0.1119	0.0186
VCI	0.2673	0.7047	0.4300	0.0769	0.0609
NDMI	−0.1795	0.1992	−0.0359	0.0696	0.0628
DEM (m)	137.1368	185.1635	149.5263	8.8959	0.0830
TWI	0	13.1843	10.1422	1.7216	0.0623
prox2river (m)	0	1000	480.3238	254.7689	0.1334
Slope (°)	0.2356	1.7469	0.7553	0.2212	0.2163

Note: Correlation values represent Pearson’s r with drought count. All remote sensing indices show correlations < |0.10|, indicating weak univariate relationships. SMI and NDMI show high correlation (r = 0.92) but were retained for evaluation.

**Table 3:** Binary classification performance metrics for drought detection (threshold > 1 point; test set: 2024, n = 203).

Metric	AUC	Accuracy	Balanced Accuracy	Precision	Recall	Specificity	F1-Score	FPR	FNR
Value	0.783	0.783	0.764	0.801	0.820	0.708	0.810	0.292	0.180

Note: LightGBM achieved the highest test AUC (0.783), accuracy (78.3%), balanced accuracy (76.4%), and F1-score (0.810), outperforming XGBoost (AUC = 0.769) and Random Forest (AUC = 0.712). The majority class baseline achieves 65.5% accuracy and 100% recall, highlighting the class imbalance (65.5% drought class in test set).

**Table 4:** Binary classification performance under spatial holdout validation (leave-location-out, 5-fold spatial blocking).

Model	AUC	Accuracy	Balanced Accuracy	Precision	Recall	F1-Score
LightGBM	0.714	0.679	0.651	0.712	0.723	0.717
XGBoost	0.698	0.662	0.638	0.695	0.708	0.701
Random Forest	0.641	0.623	0.592	0.658	0.671	0.664

Note: Performance drops substantially under spatial holdout compared to temporal validation (Table 3), indicating that some predictive skill is attributable to cell-specific spatial fingerprints rather than generalizable drought prediction. LightGBM remains the best-performing model but with AUC reduced from 0.783 to 0.714.

**Table 5:** Per-class precision, recall, and F1-scores for LightGBM on the 2024 test set (n = 203) for five-class severity classification.

Class	Precision	Recall	F1-Score	Support
Very Low	0.769	0.714	0.741	70
Low	0.552	0.627	0.587	51
Moderate	0.543	0.485	0.512	66
High	0.308	0.333	0.320	12
Severe	0.167	0.250	0.200	4

Note: Overall macro-average F1 = 0.472, weighted-average F1 = 0.591. Performance declines substantially for rare classes (High, Severe) due to limited training samples (n ≤ 12 in test set). The Very Low class shows the best performance due to its majority representation.

**Table 6:** SHAP feature importance ranking for drought risk assessment in Maha Sarakham Province (based on LightGBM model retrained without NSMI).

Rank	Feature	Mean SHAP Value	Relative Importance (%)	Cumulative (%)
1	slope	1.4662	26.4820	26.4820
2	dem	0.8870	16.0220	42.5040
3	prox2river	0.8549	15.4419	57.9459
4	twi	0.6762	12.2138	70.1597
5	ndmi	0.4808	8.6846	78.8443
6	ndvi	0.4165	7.5230	86.3673
7	mndwi	0.2941	5.3113	91.6786
8	smi	0.2658	4.8015	96.4801
9	vci	0.1949	3.5199	100.0000

Note: Static spatial variables collectively account for 76.1% of total SHAP importance (slope 28.5% + DEM 18.2% + TWI 16.3% + prox2river 13.1%), while remote sensing indices contribute 23.9% (NDVI 7.8% + SMI 6.5% + MNDWI 4.3% + NDMI 3.1% + VCI 2.2%). NSMI was removed from the analysis due to perfect collinearity with SMI.

**Table 7:** Label permutation test results (100 permutations, LightGBM).

Metric	Permutation Mean	Permutation SD	95% CI Lower	95% CI Upper	Actual Model	Z-Score
AUC	0.512	0.034	0.445	0.579	0.783	7.97
Accuracy	0.561	0.028	0.506	0.616	0.783	7.93
Balanced Accuracy	0.500	0.019	0.463	0.537	0.764	13.89

Note: Z-score = (Actual − Permutation Mean)/Permutation SD. All actual model performance metrics fall well outside the 95% confidence intervals of the permutation distributions, confirming statistically significant predictive signal (*p* < 0.01 for all metrics).

**Table 8:** Benchmark comparison of machine learning model performance for drought prediction across regions, including the current study.

Study	Region	Method	Best Performance	Validation
Park et al. [55]	South Korea	Random Forest	AUC = 0.92–0.95	Temporal
Dikshit et al. [56]	Australia	XGBoost	AUC = 0.91–0.94	Spatial-temporal
Hao et al. [57]	China	LightGBM	R^2^ = 0.72–0.78	Temporal
This study	Thailand	LightGBM	AUC = 0.783 (test), 0.714 (spatial)	Temporal + Spatial

## Data Availability

The original data presented in this study are openly available at https://doi.org/10.5281/zenodo.21257445. The repository contains the corrected dataset, analysis code, and additional materials.
